# Molecular characterization of methicillin-resistant *Staphylococcus aureus* (MRSA) nasal colonization and infection isolates in a Veterans Affairs hospital

**DOI:** 10.1186/s13756-015-0048-5

**Published:** 2015-04-02

**Authors:** Kalyani E Eko, Brett M Forshey, Margaret Carrel, Marin L Schweizer, Eli N Perencevich, Tara C Smith

**Affiliations:** Department of Epidemiology, University of Iowa College of Public Health, Iowa City, IA 52246 USA; Center for Comprehensive Access & Delivery Research and Evaluation (CADRE), Iowa City VA Health Care System, Iowa City, IA 52246 USA; Department of Internal Medicine, University of Iowa Carver College of Medicine, Iowa City, IA 52246 USA; Department of Geographical & Sustainability Sciences, University of Iowa, Iowa City, IA 52242 USA; Department of Biostatistics, Environmental Health Sciences and Epidemiology, College of Public Health, Kent State University, Kent, OH 44242 USA

**Keywords:** *Staphylococcus aureus*, MRSA, Hospital acquired infections, Infection control

## Abstract

**Background:**

Nasal colonization with methicillin-resistant *Staphylococcus aureus* (MRSA) is associated with increased infection risk, yet colonization and infection isolates are rarely compared within the same study. The objectives of this study were to compare colonization and infection isolates from a Veterans Administration hospital in Iowa, and to determine the prevalence of livestock-associated MRSA (LA-MRSA) colonization and infection in a state with high livestock density.

**Methods:**

All patients with available MRSA isolates collected through routine nasal screening (73%; n = 397) and from infections (27%; n = 148) between December 2010 and August 2012 were included and tested for *spa* type and presence of PVL and *mec*A genes. Clinical isolates were tested for antibiotic resistance patterns. Paired colonization and infection isolates were compared for genetic and phenotypic congruity.

**Results:**

The most common *spa* types were t002 (and other CC5-associated strains; 65%) and t008 (and other CC8-associated strains; 20%). No classic LA-MRSA *spa* types were identified. CC5-associated strains were less likely to be associated with infections (22%; 77/353) compared with CC8-associated strains (49%; 53/109). MRSA colonization was more common among patients with infections (71%) compared with the general screening population (7%). In most cases (82%; 28/34), paired colonization and infection isolates were genetically and phenotypically indistinguishable.

**Conclusions:**

Our data demonstrate a direct link between antecedent nasal colonization and subsequent MRSA infection. Further, our data indicate variability in colonization and infection efficiency among MRSA genotypes, which points to the need to define the molecular determinants underlying emergence of *S. aureus* strains in the community and nosocomial setting.

## Introduction

As the leading cause of multi-drug resistant nosocomial infections in the U.S. [1], methicillin-resistant *Staphylococcus aureus* (MRSA) is often responsible for ventilator-associated pneumonia, septicemia associated with venous catheters, and surgical site infections [2]. Treatment is complicated by resistance to multiple antimicrobial compounds. In addition to infections, MRSA can be found to asymptomatically colonize sites such as the nose and throat. The prevalence of MRSA nasal colonization in the general U.S. population is approximately 1 – 2% [3], but can exceed 13% among admitted Veterans Affairs (VA) hospital patients [2] and 10 to 15% of patients within U.S. acute-care hospitals and intensive care units [4].

MRSA colonization is associated with increased risk for MRSA infection [5], with an estimated 4-fold increase in the odds of infection [6]. In an effort to control the spread of MRSA, the VA health care system implemented a nationwide surveillance program [2,7], which included universal nasal surveillance for MRSA colonization, contact precautions for patients carrying MRSA, and emphasis on proper hand hygiene.

The epidemiology of MRSA has evolved considerably. Originally, MRSA strains could be traced back to a hospital or clinic source. More recently, MRSA strains have been recognized within the community setting and in animal production workers. In food-producing states like Iowa, the emergence of livestock-associated MRSA (LA-MRSA) strains is of particular concern. Residents often live in close proximately to large scale livestock production facilities, such as confined animal feeding operations (CAFOs), where LA-MRSA and other MRSA strains have been found to colonize swine [8,9]. Our previous study found that geographic proximity to large swine populations was associated with increased risk of MRSA colonization among VA patients in Iowa [10].

Although several studies have pointed to a link between MRSA colonization and increased risk for invasive infection, few studies have directly compared colonization and infection isolates [11]. More detailed studies are necessary to define the link between colonization and infection. In this study, we characterized MRSA isolates from the Iowa City Veterans Affairs Health Care System (ICVAHCS), collected through routine screening for nasal colonization and from clinical isolates. Our objectives were to compare the molecular and phenotypic characteristics between colonization and clinical isolates at the population and individual level, and to explore molecular and phenotypic markers that might be indicative of livestock origin.

## Methods

### Human subjects

Both the ICVAHCS Research and Development Committee and the University of Iowa’s Institutional Review Board approved this study.

### Sample collection and culture

All patients with available MRSA isolates collected between December 2010 and August 2012 and stored at ICVAHCS were analyzed. Samples had been collected through routine nasal screening and from patient wound sites and bodily fluids. During this time period, all patients were screened for MRSA nasal colonization at hospital admission, unit transfer and hospital discharge. Not all *S. aureus* isolates were saved for analysis. Preference was given to clinically significant or molecularly interesting isolates, such as *mec*A negative MRSA isolates. Samples were plated on blood agar and identified as *S. aureus* based on conventional methods. Samples were further identified as MRSA based on polymerase chain reaction, as described elsewhere [12], and then banked for future analyses. MRSA samples were stored at -80°C ICVAHCS were stored in vials at until September 2013, when they were plated on Columbia agar plates containing 5% sheep blood, colistin, and nalidixic acid (CNA plates; Remel) and incubated overnight at the 37°C and 5% CO_2_. All samples initially identified as positive for the *mec*A gene were re-tested for confirmation.

### Molecular testing

Genomic DNA was extracted and subjected to molecular typing. Positive and negative controls were used during all molecular experiments. The presence of *scn*, Panton-Valentine leukocidin (PVL), and *mec*A genes were determined by polymerase chain reaction (PCR) [13,14]. The absence of *scn* is considered to be a marker of LA-MRSA [15,16].

*spa* typing was performed using an alternate forward primer as described [17] and a reverse primer as specified on the Ridom website (http://www.ridom.de/doc/Ridom_spa_sequencing.pdf). *spa* types were determined through the Ridom StaphType software package (Ridom GmbH, Wurzburg, Germany). MRSA isolates were genetically clustered based upon *spa* type according to the Based upon Repeat Pattern (BURP) algorithm [18]. Isolates were placed into cluster complexes according to relatedness (i.e. within 6 steps) to more common *spa* types, such as t002 and t008. Where possible, placement into a particular cluster complex was evaluated based on typical *spa* type-multilocus sequence type (MLST) associations reported in the literature (eg, t045 and t002 are typically associated with CC5; [19] and the Ridom website http://spa.ridom.de/spatypes.shtml). *spa* types associated with CC398 and CC9 were considered LA-MRSA.

### Antimicrobial susceptibility testing

Antimicrobial susceptibility testing (AST) results were available for a subset of patient samples (129/545; 23.7%), predominantly for infections requiring clinical treatment. Isolates were subject to an AST panel as described by the Clinical and Laboratory Standards Institute [20]. Isolates were tested for susceptibility to the following antibiotics: benzylpenicillin, cefoxitin, oxacillin, tetracycline, gentamicin, linezolid, tigecycline, quinupristin-dalfopristin, trimethoprim-sulphamethoxazole, vancomycin, fluoroquinolones (ciprofloxacin, levofloxacin, moxifloxacin), clindamycin, clindamycin (inducible resistance), and erythromycin.

As tetracycline resistance is a possible marker for livestock-associated strains [21] and t002 isolates have been found in North American swine [9,22-24], *spa* type t002 isolates were further analyzed for susceptibility to tetracycline.

### Paired samples

To determine the contribution of nasal carriage strains to observed infections, we compared the molecular and AST (where available) characteristics of paired nasal swab isolates and infection isolates. Sample pairs where an infection isolate was collected within 30 days of a screening swab from the same patient were included in the analysis.

### Data collection and analysis

Epidemiological data was maintained at ICVAHCS, including age, sex, home address, race/ethnicity, specimen type, and specimen collection date. All samples and data were de-identified prior to analysis. All data analysis was conducted in SAS version 9.3 (Cary, NC).

### Geocoding and mapping

Each sample was linked to a patient address and geocoded to a latitude/longitude coordinate. Samples with matched addresses were then mapped over the boundaries of the state of Iowa and Census defined urban areas in the state, stratified by *spa* type.

## Results

### Sample sources and patient demographics

In total, 545 samples (98.0%; 545/556) were confirmed as positive for *mec*A and were subjected to further analysis. Overall, the majority of samples (397/545; 72.8%) were collected through routine nasal screening for MRSA colonization upon hospital admission, while the rest (148; 27.2%) were collected from infections. Data was available for 85 infection isolates with specimens collected from wounds or abscesses [n = 48], blood [n = 17], urine [n = 10], sputum [n = 7]), bone [n = 2], and synovial fluid [n = 1]. Personally identifiable information could be linked to 374 (68.6%) samples from 245 individuals. For most individuals (181; 73.9%) only 1 sample was analyzed, whereas for 64 individuals (26.1%), 2 or more samples were analyzed. The majority of patients with identifiable information were white (217/230 with reported race; 94.3%) and male (232/245; 94.7%). The median age was 65 years (range 23–98 years).

### Antibiotic resistance/susceptibility patterns

In total, 129 samples were tested for antibiotic susceptibility patterns; these samples were predominantly collected from infections (127; 98.5%). Nearly all tested isolates were resistant to beta-lactam antibiotics (Table 1). Fewer isolates were resistant to erythromycin (89%), fluoroquinolones (ciprofloxacin, levofloxacin, and moxifloxacin [63%]), clindamycin (47%), inducible clindamycin (26%), and gentamicin (2%). Six percent of the isolates tested were resistant to tetracycline. None of the isolates were resistant to linezolid, nitrofurantoin, quinupristin-dalfopristin, tigecycline, trimethoprim-sulphamethoxazole, or vancomycin.

**Table 1:** **Antibiotic resistance patterns of MRSA isolates (n = 129)**

**Antibiotic**	**All isolates (n = 129)**	**ST5-associated** ***spa*** **types**		**ST8-associated** ***spa*** **types**	
		**All ST5**	**t002**	**All ST8**	**t008**
		**(n = 69)**	**(n = 52)**	**(n = 46)**	**(n = 43)**
Benzylpenicillin	129 (100%)	69 (100%)	52 (100%)	46 (100%)	43 (100%)
Oxacillin	126 (98%)	66 (96%)	49 (94%)	46 (100%)	43 (100%)
Cefoxitin	126 (98%)	66 (96%)	49 (94%)	46 (100%)	43 (100%)
Erythromycin	115 (89%)	60 (87%)	49 (94%)	45 (98%)	42 (98%)
Ciprofloxacin	81 (63%)	59 (86%)	49 (94%)	20 (43%)	20 (47%)
Levofloxacin	81 (63%)	59 (86%)	49 (94%)	20 (43%)	20 (47%)
Moxifloxacin	81 (63%)	59 (86%)	48 (92%)	20 (43%)	20 (47%)
Clindamycin	61 (47%)	58 (84%)	48 (92%)	2 (4%)	2 (5%)
Inducible clindamycin	33 (26%)	33 (48%)	31 (60%)	0 (0%)	0 (0%)
Tetracycline	8 (6%)	4 (6%)	2 (4%)	3 (7%)	2 (5%)
Gentamicin	3 (2%)	2 (3%)	0 (0%)	1 (2%)	1 (2%)

No isolates were resistant to linezolid, nitrofurantoin, quinupristin-dalfopristin, tigecycline, trimethoprim-sulphamethoxazole, and vancomycin.

### Molecular characteristics

A *spa* type was defined for 93.6% (510/545) of isolates included in the study. The most common *spa* types were t002 (272; 53.3%), t008 (101; 19.8%), t045 (13; 2.6%), t088 (12; 2.4%), t242 (11; 2.2%), t11962 (9; 1.8%), t688 (8; 1.6%), and t216 (6; 1.2%). All other *spa* types individually constituted less than 1% of the total. The majority of isolates had *spa* types that have been associated with CC5 (typically considered HA-MRSA), based on associations often reported in the literature [19] or based on genetic relatedness to t002 (353; 64.8%). After CC5, the most common genetic type was CC8 (typically considered CA-MRSA), based on the literature or on relatedness to t008 (109; 20.0%). No *spa* types typically associated with CC398 or CC9 (typically considered livestock-associated strains) were identified.

PVL was detected in 23.7% (129/545) of isolates. For CC5-associated *spa* types, only 3.4% (12/353, including 6/272 for t002) were PVL-positive, whereas for CC8-associated *spa* types, 90.8% (99/109, including 93/102 for t008) were PVL-positive.

Our previous studies have demonstrated a high prevalence of MRSA on swine farms in Iowa [8,9]. Notably, not all MRSA strains identified on swine farms belonged to typical LA- MRSA sequence types. To determine if swine operations could be a source for MRSA colonization and infections in humans, we further analyzed isolates for markers of livestock origin. Based on our previous observation of t002 in livestock, we examined isolates with the t002 *spa* type (n = 272) for tetracycline resistance and the absence of the *scn* gene, both considered markers of livestock origin [15,21]. Eight t002 isolates (2.9%) were found to be tetracycline resistant, and the *scn* gene was absent from 7 (2.6%) t002 isolates; no t002 isolates had both of these markers of livestock-association.

There was considerable variation among *spa* types in relative representation among screening isolates compared with infection isolates. CC5-associated isolates comprised 69.5% of screening strains (276/397, including 214/397 [53.9%] for t002 specifically), while CC8-associated isolates comprised 14.1% (56/397, including 52/397 [13.1%] for t008 specifically). In contrast, among infection isolates, the disparity was much smaller: CC5-associated strains comprised 52.0% of infection isolates (77/148, including 39.2% [58/148] for t002 specifically), whereas CC8-associated strains comprised 35.8% (53/148, including 33.1% [49/148] for t008 specifically).

### Spatial analysis of colonization isolates

**Figure 1:**
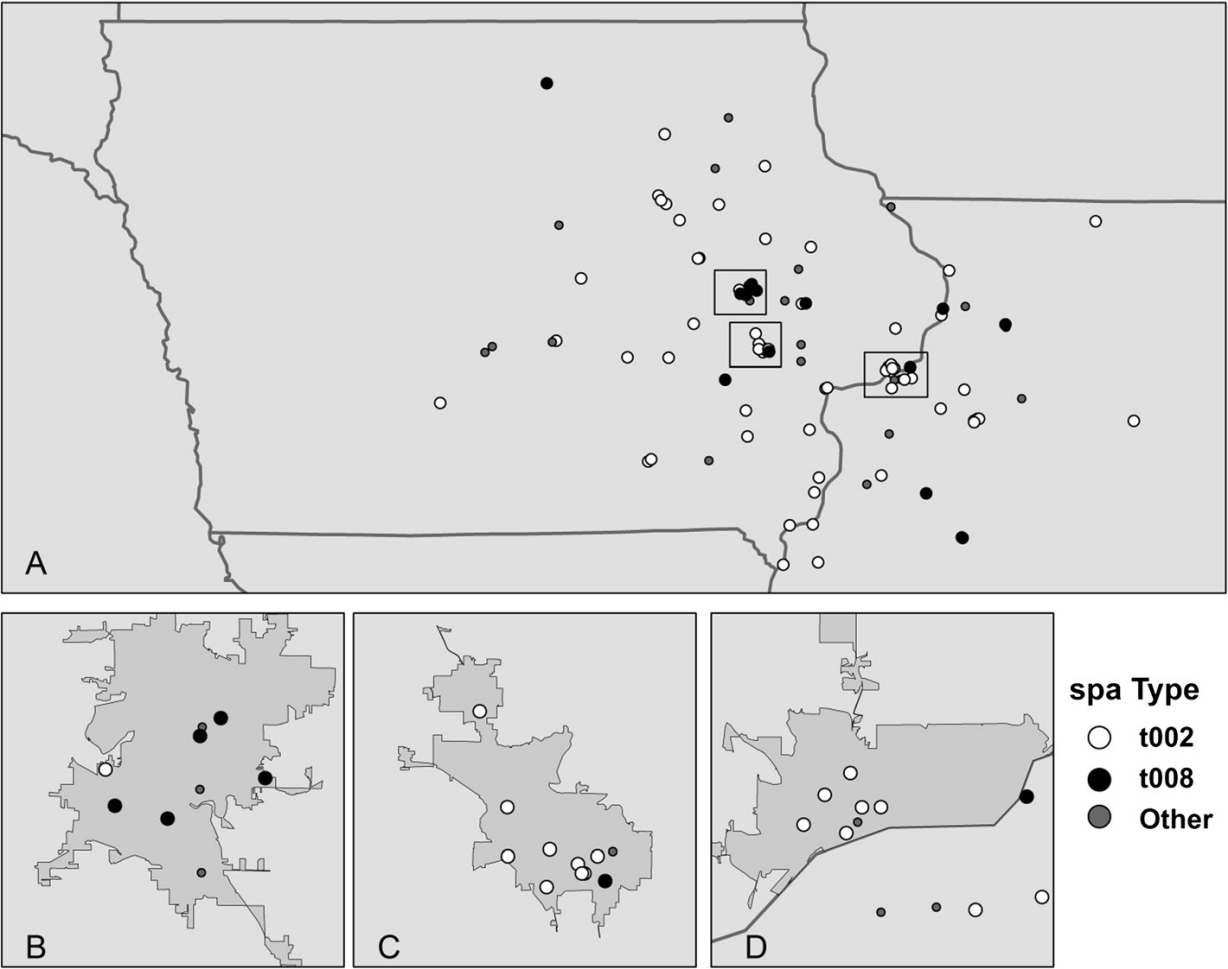
**Residential locations of patients with MRSA nasal isolates collected during admission screening at the ICVAHCS. (A)**. Urban areas of Cedar Rapids **(B)**, Iowa City/Coralville **(C)**, and the Quad Cities **(D)** are shown in greater detail.

To determine the spatial heterogeneity of MRSA isolates, *spa* types of colonization isolates were mapped to patients’ home addresses (n = 115) (Figure 1A). Of these, 15 (13%) were t008, 70 (61%) were t002, and 30 (26%) were classified as “other”, consistent with the overall frequency of *spa* types among colonization isolates (13%, 54%, and 33% for t008, t002, and “other”, respectively). No duplicate samples (i.e. two from one patient) were included in the geocoded dataset. The overall spatial pattern reflects the typical service area of the ICVAHCS, drawing primarily from Eastern Iowa but also from patients located in other areas of Iowa and adjacent states. A cluster of t008 isolates is observed in the patients residing in the Cedar Rapids urban area, while t002 is more prevalent in Iowa City/Coralville and the Quad Cities (Figure 1B-D). The dates of isolation for the t008 samples in Cedar Rapids span over 9 months, so are not reflective of a single outbreak.

### Comparison of nasal colonization and infection isolates

To determine the relationship between nasal carriage and infection, we compared screening results for patients with MRSA infections with the screening results of the patient population at-large. In total, 28 patients with MRSA infections could be linked to screening results within 30 days prior to the infection sample. Of these, 20 were MRSA-positive at admission screening (71%), compared with 7% for the ICVAHCS overall during a similar time period [10]. We also were able to identify 34 patients who had both an MRSA infection isolate available for testing and a MRSA isolate from recent admission screening. Of these, 28 (82%) had the same molecular characteristics, including spa type, for the screening and infection isolates (Table 2). Additionally, for one other patient the spa types of the colonizing (t024) and infecting strains (t008) were genetically closely related, separated only by a single deletion/insertion event.

**Table 2:** **Comparison of** ***spa*** **types between paired screening and infection isolates**

**Screen isolate** ***spa*** **type**	**Infection isolate** ***spa*** **type**	**N**
t002	t002	15
t008	t008	8
N/A	t002	2
t12159	t12159	1
t088	t088	1
t216	t216	1
t051	t051	1
t242	t242	1
t024	t008	1
t306	N/A	1
t002	t686	1
t2049	t126	1

N/A indicates that a *spa* type was not identified.

## Discussion

Our results support previous studies [4,25-27] that demonstrate a link between nasal colonization and subsequent clinical infections with MRSA, as colonization upon hospital admission was much higher among patients with MRSA infections than in the general VA patient population. The molecular analysis provides further context: for most patients with clinical infections, who had been found to be colonized upon admission, paired MRSA isolates were largely genetically and phenotypically indistinguishable. These data suggest that colonization and infection are often directly linked, and that the effect is not primarily driven acquisition in hospital settings. Further, our data suggest that the efficiency of colonization and infection may vary among genetic types. Specifically, CC8-associated *spa* types made up only 13% of colonization isolates, yet they made up more than 30% of infection isolates (i.e., a ratio of 1 infection isolate to every colonization isolate). In comparison, CC5-associated *spa* types made up more than nearly 70% of colonization isolates, yet accounted for a similar percent of infection isolates as ST8 (i.e., there was 1 infection isolate to every 4 colonization isolates).

Consistent with a recent nationwide study of clinical MRSA isolates [28], we found t002 (typically considered HA-MRSA) and t008 (typically considered CA-MRSA) to be the most common *spa* types associated with infections. In that study, t008 (51%) was the most common, followed by t002 (18%), compared with 33% and 39%, respectively, in our study. These data further indicate that USA300/t008/CA-MRSA strains are supplanting traditional HA-MRSA strains in nosocomial settings.

We observed different geographical clustering of *spa* type t002 versus t008 in urban areas of Iowa. Although the exact mechanism for this clustering is unknown, it is possible that other factors associated with MRSA carriage may account for this indication of geographical grouping. For instance, a common community reservoir of community-associated *spa* type t008 may be shared by veterans in the city of Cedar Rapids. These types of reservoirs have been identified in MRSA outbreak situations but would be difficult to identify for MRSA colonization [29].

Our previous study indicated that proximity to high density swine farms is associated with higher risk for MRSA colonization among VA patients [10]. However, we did not find evidence for a significant number of LA-MRSA strains. Specifically, we did not find any typical livestock associated *spa* types (CC398 or ST9), tetracycline resistance was uncommon, and few isolates were negative for the *scn* gene. Similarly, studies in North Carolina [30] and Pennsylvania found that proximity to livestock operations and swine manure application to crop fields is associated with increased odds for MRSA infection [31]. Yet, in those studies, no typical LA-MRSA *spa* types were found, and few isolates were *scn*-negative [30,32]. Our previous results from Iowa, Ohio, and Connecticut have shown that while CC398 is the most common sequence type [8,9,22], other genetic types have also been found to colonize swine [22-24,33]. Taken together, these results suggest that swine may be a source of non-LA-MRSA colonization and infections in humans.

Through this study, the surveillance and molecular characterization of a convenience sample of MRSA isolates within the ICVAHCS provides for a better picture of MRSA epidemiology within similar hospital settings. However, a possible limitation of our study includes the degree to which our findings can be generalized to other populations and institutions. For example, our study was predominantly older and male, and Diekema et al found that USA100/t002 was more commonly isolated from older patients (> = 65 yrs) compared with USA300/t008 [28]. Additionally, although our study included both nasal screening and clinically significant isolates that were obtained from veterans admitted to the ICVAHCS, it is possible that our convenience sample may not include some patients who were colonized or infected at time of admission, whose isolates were not saved, or who were colonized at another anatomical site. Thus, our results must be interpreted with caution, since the associations between infection and *spa* type may be alternatively explained by infecting isolates from an outbreak being preferentially saved over infecting isolates from a non-outbreak *spa* type. Furthermore, some patients were admitted and screened multiple times, generating multiple observations. Another possible limitation of our analysis was reliance on clustering of *spa* types, rather than multilocus sequence typing (MLST). However, a high concordance between *spa* type and MLST groupings has been demonstrated in several studies [34,35].

## Conclusions

Our data confirm the link between nasal colonization and clinical MRSA infection and point to the need to better understand the molecular determinants underlying *S. aureus* emergence, including factors that enhance colonization and infection. Our results indicate that USA300 (e.g., t008) emergence may be driven in part by increased infectivity, rather than increased ability to colonize individuals necessarily. Further, the link between nasal colonization strains and clinical infection strains suggest the need to continue to explore active surveillance and mupirocin decolonization as a means to reduce the burden from MRSA infections [7]. Livestock-associated MRSA does not appear to contribute significantly to the prevalence of MRSA colonization or the burden of MRSA infections in ICVAHCS, despite the high density of livestock in the region.
